# Study protocol for a randomized clinical trial to evaluate the effect of the use of Xylitol gum in the prevention of caries lesions in children living in Ladakh—the *Ca*ries *Pre*vention *X*ylitol in *Ch*ildren (CaPreXCh) trial

**DOI:** 10.1186/s13063-021-05828-y

**Published:** 2021-12-04

**Authors:** Maria Grazia Cagetti, Fabio Cocco, Ezio Calzavara, Davide Augello, Phunchok Zangpoo, Guglielmo Campus

**Affiliations:** 1 Department of Biomedical, Surgical and Dental Sciences, University of Milan, Via Beldiletto 1, 20142 Milan, Italy; 2 Department of Medicine, Surgery and Experimental Sciences, School of Dentistry, University of Sassari, Viale San Pietro, 07100 Sassari, Italy; 3 Italian Association of Dentists (ANDI), Rome, Italy; 4 Community Health Center, Dental Service, Padum, Ladakh, India; 5 Department of Restorative, Preventive and Pediatric Dentistry, University of Bern, Freiburgstrasse 7, 3010 Bern, CH Switzerland; 6 Department of Pediatric, Preventive Dentistry and Orthodontics, School of Dentistry, Sechenov University, 119, Moscow, 119991 Russia

## Abstract

**Background:**

Xylitol use is reported to be able to reduce dental plaque amount and cariogenic bacteria and, as a consequence, the caries increment. Only few data on the oral health of Ladakh’s population are available. The aim of the present protocol will be to record the caries prevalence of primary and permanent molars of schoolchildren living in Ladakh and to implement a school-based Xylitol programme, named the *Ca*ries *Pre*vention *X*ylitol in *Ch*ildren (CaPreXCh) trial, using chewing gums.

**Methods:**

The protocol is designed as a triple-blind randomized, controlled, parallel-group clinical trial in children aged 5–14 years. The study should have been carried out from August 2021 to August 2024 in Zanskar Valley (Ladakh), but the COVID-19 pandemic does not allow today to make predictions on the exact start. Participants will be randomly allocated into two groups: subjects who will receive a chewing gum with Xylitol (70% w/v) as only sweetener, and those who will receive a sugared chewing gum containing Maltitol (23% w/v). The subjects will be instructed to chew a total of 6 pellets for 5 min divided into 3 intakes a day (2 in the morning, 2 after the midday meal and 2 in the afternoon) for one school year. Clinical examination will comprise an oral examination in which caries index (ICDAS scores), bleeding on probing and plaque pH evaluation after sucrose challenge will be recorded at baseline (*t*_0_); the clinical examination will be repeated after 12 months since the beginning of the chewing gum administration period (*t*_1_), after another 12-month period (*t*_2_) and finally after further 12 months (*t*_3_) (24 months from the end of the chewing gum use). The primary outcome will be the caries increment measured both at enamel and dentinal levels on primary and permanent molars. Data analysis will be conducted through Kaplan-Meyer graphs to evaluate caries increment. A comparison of the methods will be carried out with Cox regression with shared frailty. The net caries increment for initial, moderate and severe caries levels, using ICDAS (Δ-initial, Δ-moderate and Δ-severe), will be calculated.

**Discussion:**

This trial will be the first trial conducted in India assessing the efficacy of a school-based caries preventive programme through the use of chewing gum containing only Xylitol as a sweetener. The findings could help strengthen the evidence for the efficacy of Xylitol use in community-based caries prevention programmes in children.

**Trial registration:**

Clinical trials.govNCT04420780. Registered on June 9, 2020

**Supplementary Information:**

The online version contains supplementary material available at 10.1186/s13063-021-05828-y.

## Introduction

Ladakh is a region administered by India, covering an area slightly larger than Croatia; it is part of the larger region of Kashmir and Jammu, which has been the subject of dispute between India, Pakistan and China since 1947. The Indian Government reports Ladakh to be one of the districts that are below health service and condition standards [1]. The complex terrain and the high altitude, above 9000 ft, is responsible for the cold weather, a limited diet, a limited availability of drinking water and the poor socioeconomic conditions, factors affecting the population’s wellbeing [2–4].

The Italian National Association of Dentists Foundation (ANDI Foundation) has among its main aims that of improving the quality of life of populations with socioeconomic inequalities in various parts of the world, as the population living in the Zanskar Valley of Ladakh. Dental care is currently limited to small dental clinics, as at the medical centre in Padum (CHC, community health centre). During summertime, when the valley is accessible, teams of volunteers from the Italian National Dentistry Association (ANDI) Foundation work as dentists at the Padum’s facility and promote oral health in schools.

Oral health data on the population living in Ladakh are poor. A recent survey performed on schoolchildren living in Ladakh showed that caries was almost ubiquitarian with only 10% of caries-free children [5]. Caries severity, in both primary and permanent dentitions, was statistically significantly related to gender, waist circumference, body mass index (BMI), oral hygiene frequency and self-reported chewing problems. An increasing relative risk for caries in permanent dentition compared to caries-free subjects was observed in children with a low BMI [5].

The frequent intake of fermentable carbohydrates with the diet favours a high plaque cariogenicity, increasing the possibility of demineralization of enamel and dentin; the acidic environment of the plaque in turn promotes the growth of cariogenic bacteria, further increasing the caries risk [6]. As a consequence, different preventive approaches of caries prevention have focused on the reduction of sugar intake and its replacement with non-fermentable sweeteners, such as Xylitol. Polyols were proposed as an anti-caries agent since the 1970s, delivered mostly through chewing gum. Xylitol-based caries prevention programmes have been carried out in children and adults, showing the capacity of the polyol in reducing caries risk factors and caries incidence [7–9]. An advantage of using Xylitol in caries prevention programmes is that it does not require specifically trained personnel for its administration, since it can be easily given both at home by parents and at school by teachers even in less served areas of the earth.

Although encouraging results were obtained in children in ten RCT studies, the quality of evidence was rated as very low [10], calling for the need of well-conducted and methodological sound trials. Moreover, the use of Xylitol for caries control is considered a preventive method that might encounter dietary-cultural obstacles, especially in countries where polyol and or chewing gum use are not frequently consumed, and this aspect needs also to be further investigated.

Recently, a chewing gum sweetened with Xylitol only was proposed. A preliminary study showed that this product is able to increase significantly the Xylitol concentration in saliva compared to the level obtained using a chewing gum containing different polyols. Furthermore, the chewing gum also demonstrated to have the capacity of reducing bacterial strains related to caries and periodontal disease [11].

The aim of the present protocol will be to register the caries prevalence of schoolchildren living in Ladakh and to implement a school-based Xylitol programme using chewing gums in order to reduce caries incidence.

## Methods

### Trial design

**Fig. 1:**
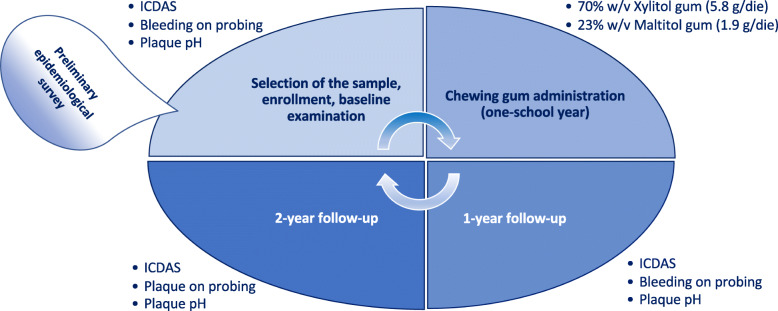
The flowchart of the study design

This is the third and final version of the protocol (edited on the 15th of May 2021). A superiority trial is designed to detect a difference between treatments as a triple-blind randomized, controlled, parallel-group clinical trial. Two groups will be compared: subjects who will receive a Xylitol chewing gum with the polyol as the only sweetener (70% w/v), and those who will receive sugared gum containing a 23% (w/v) Maltitol. The flowchart of the study design is displayed in Fig. 1.

The investigation will have an experimental period of one school year (approximately 9 months in total). The study will gain the approval of all the schools’ authorities. Due to language problems and the low literacy rate of the population, parents’ or guardians’ verbal approval will be obtained for children’s participation.

The trial—Caries Prevention Xylitol in Children (CaPreXCh)—was registered with ClinicalTrial.gov (NCT04420780) and is currently in the no-recruiting phase. The Standard Protocol Items for Clinical Trials (SPIRIT) was used to guide the present protocol as detailed in online supplementary appendix (Additional file 1).

The study should have been carried out from August 2021 to August 2024 in Zanskar Valley (Ladakh), but due to the persistence of the COVID-19 pandemic, it is not possible to predict the real beginning of the trial.

### Participants, interventions and outcomes

#### Setting

The study will be conducted in the Zanskar Valley (Ladakh). School classes of subjects aged 5–14 years old will be randomly selected from a list of classes in the selected schools.

In 2018, the total schoolchildren population in the Zanskar Valley aged 5–17 years amounted to 2407 children.

#### Eligibility: inclusion and exclusion criteria

The inclusion criteria will consider the following:

a) To be 5–14 years old

b) To be in good general health

The exclusion criteria will consider the following:

a) Children who refuse to participate in the research; parents/guardians who refuse the participation of their children to the trial

b) Children who present systemic conditions or chronic diseases that require differentiated care and follow-up

#### Preliminary epidemiological survey

A team of four dentists received training to diagnose caries lesions using the WHO-DMFT index for permanent teeth and the dmft for primary teeth. The team received training, and inter-examiner reliability was assessed before the start of the study; sensitivity, specificity, percentage agreement and kappa statistics were recorded. Inter-examiner reliability ranged from 0.75 to 0.84 (K-Cohen) for sound teeth and from 0.82 to 0.88 for caries lesions. Intra-examiner reliability ranged from 0.82 to 0.90 for sound teeth and from 0.84 to 0.91 for caries lesions. Dental caries prevalence (dt/DT) and severity (number of lesions) were recorded for caries at the dentinal lesion level [12]. Every subject was examined using a plain mirror (Hahnenkratt, Königsbach, Germany) and the WHO CPI ballpoint probe (Asa-Dental, Milan, Italy), under standard light. No bitewing radiographs or fibre-optic trans-illumination were used.

An ad hoc prepared questionnaire assessed general health, eating habits, oral hygiene and the self-perception of oral conditions, based on previous surveys [12, 13]. The height (cm) and the weight (kg) were measured and the body mass index (BMI) was calculated. The waist circumference was also measured.

Caries was almost ubiquitarian with only 10.04% of caries-free children (dt/DT = 0). Caries severity, in both primary and permanent dentitions, was statistically significantly related to gender and waist circumference (*p* < 0.01). This preliminary epidemiological survey will allow to design the intervention trial.

### Interventions

After obtaining actual caries data, schools with similar numbers of schoolchildren (at least 300 subjects per school) will be selected for the preventive project. The participants will be randomized, taking as cluster the school class, allocating each class into one of the two groups of the study (Fig. 1):

(1) The first group (Xyl) will receive sugar-free gums containing 70% (w/v) Xylitol as the only sweetener

(2) The second group (Pol) will receive reduced sugared gums containing 23% (w/v) Maltitol

Both groups of children will receive a total of 6 pellets of chewing gum a day during a period of one school year (about 9 months). All chewing gums will be produced and supplied by Perfetti Van Melle SpA (Lainate, Italy). All types of chewing gums will weigh 1.4 g each and will be identical in colour, shape and taste.

#### Clinical examination

Clinical examination, coordinated by E.C. as responsible of the coordinating centre, will comprise an oral examination (caries index, bleeding on probing recording) and an evaluation of plaque pH fluctuation after the sucrose challenge. Bleeding on probing will be used as a proxy of plaque presence, since there is a causal relationship between bleeding on probing and amount of plaque [13, 14].

The subjects will be examined using a mouth mirror, a ball-ended probe and artificial light. Caries registration will be performed with regard to the first and second primary, and first and second permanent, molars. The International Caries Detection and Assessment System (ICDAS) will be used to record caries at tooth level as initial, moderate or severe lesions, the number of filled teeth and the missing teeth for caries [15, 16]. Initial caries lesion can be defined as a primary lesion, which has not reached the stage of an established lesion with cavitation (ICDAS scores 1 and 2). Moderate caries lesions are defined as white or brown spot lesion with localized enamel breakdown or an underlying dentine shadow without visible dentine exposure (ICDAS scores 3 and 4). Extensive caries lesions are defined as a distinct cavity in opaque or discoloured enamel with visible dentine (ICDAS scores 5 and 6) [9, 15, 16]. The bleeding on probing score, as the percentage of periodontal site bleeding, will be registered in all subjects.

#### Plaque pH measurements

Interproximal plaque pH, as an interim indicator, will be evaluated using pH indicator strips [17, 18], which measure a pH value in the range of 4.0–7.0 (Spezialindikator, pH range 4.0–7.0; Merck, Darmstadt, Germany). The strips determine changes in plaque pH, discriminating differences at the level of 0.2–0.5 pH units, and they are easy to use. The strips will be cut into 4 pieces (approx. 2 mm in width) in order to get a strip that could be easily inserted into the interproximal space, held in situ for 10 s and its colour compared to the colour index scheme supplied by the manufacturer.

For each subject, 3 measurements will be carried out in 2 sites, between the 2nd premolar or the 2nd primary molar, if present, and the 1st molar right and left of the upper jaw. Measurements will be performed before and at 2, 5, 10, 15 and 20 min after a mouth rinse with 10% sucrose.

#### Calibration of the examiners

Four dentists will be trained and calibrated by a benchmark examiner. Baseline training will consist of a 1-day theoretical course, followed by an examination of extracted teeth, plus a session of photographs. After 2 days of the theoretical course, a clinical training will be performed. Twenty children will be examined and re-examined after 72 h. Inter-examiner reliability with the benchmark examiner will be evaluated using fixed-effects analysis of variance and intra-examiner reproducibility assessed as the percentage of agreement using Cohen’s kappa statistic [11].

#### Sample size

The sample size calculation was performed based on the primary outcome of the randomized clinical trial (caries increment at enamel and dentinal level). The calculation considered the results of a 3-year period [7, 8]. It was also taken into account an increment of approximately 10% of the sample due to some changes in the prevalence of the disease. Thus, estimating a level of significance of 0.05 with a power of 80% and a caries prevalence of about 90% [5], the number of participants was set to 409 subjects (Kelsey method), based on a 10% absolute difference between the groups, using a two-tailed test [19].

#### Treatment

P.Z. will coordinate the treatment phase of the chewing gums. The subjects of both arms will be instructed to chew a total of 6 pellets for 5 min divided into 3 intakes a day (2 in the morning, 2 after the midday meal and 2 in the afternoon) [7, 8]. Thus, the total daily intake of Xylitol in the Xyl group will be 5.8 g, whilst, in the Pol group, the amount of administered Maltitol will be 1.9 g. Subjects will be instructed to use the chewing gum immediately after the main meals and snacks. Parents/guardians will not be requested to make changes in the dietary and oral hygiene habits of their children. Tooth brushing will not be allowed for at least 1 h after the chewing gum use. All subjects will receive a fluoridated toothpaste containing 1450 μg/g NaF and a manual toothbrush, which will be substituted every 3 months throughout the entire experimental period (3 years). All children will be instructed to brush their teeth twice a day (after breakfast and dinner) for 2 min using vertical movements [20].

In order to evaluate the success of the administration of chewing gum at school, teachers will receive 1 month at a time the chewing gums necessary for that period of time and will distribute the blister packs to their school class. Children will have to return their empty blister pack when receiving that for the following month. This procedure will be repeated for all experimental (chewing) period (1-year school calendar/9 months).

### Outcomes

The primary outcome will be the caries increment measured both at enamel and dentinal levels. The secondary outcomes will be the differences obtained comparing caries increment and the variation of plaque pH in relation to the treatment used. Furthermore, a cost-effectiveness analysis will be performed using as outcome the measurement of quality-adjusted life years (QALY) [21].

### Participant timeline

**Fig. 2:**
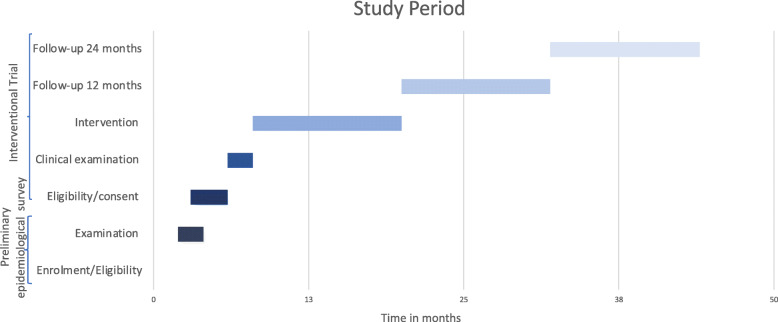
The study phases

The study should have been recruiting patients from August 2021 to September 2021; nevertheless, as previously described, COVID-19 does not allow at the moment to make predictions about it. Each subject will be enrolled for 36 months, estimating 9 months (one school year) of treatment and 27 months of follow-up. The study phases are presented in Fig. 2.

### Recruitment

The recruitment will occur in Zanskar Valley (Ladakh), in Padum city.

### Assignment of interventions

#### Allocation: sequence generation and concealment mechanism

The random list will be generated using a computer program (Microsoft Excel for Mac version 16.37); a school-class-based randomization will be carried out by G.C. in permuted blocks of 2 or 4 classes with a random variation of the blocking number and 2 groups will be created [22, 23].

The two types of chewing gums will both be supplied in plain white containers coded as ‘green’ or ‘blue’. The code will be sealed by an independent monitor and not broken until the statistical analysis will be finalized.

### Implementation

All examinations of the children enrolled will be carried out by calibrated dentists. The clinical examination of the enrolled sample will be recorded at baseline (*t*_0_), will be repeated after 12 months since the start of the chewing gum administration period (*t*_1_), after another 12 months period (*t*_2_) and finally after further 12 months (*t*_3_) (24 months from the end of the chewing gum use) (Fig. 2).

### Blinding

The study will have a triple-blind design: children, parents/guardians and teachers responsible for the treatment; dentists who will evaluate the outcomes; and the statistician who will analyse data will be blinded to the allocation group of the participants.

### Data collection, management, and analysis

The follow-up assessments will be performed by calibrated blinded dentists. The clinical data will be recorded on electronic sheets organized on PageMaker and then transferred into Microsoft Excel Software. The data management team is formed by two authors (F.C., G.C.). Data will be cleaned, deleting those revealing the participants’ identities, and they will be shared in a public repository at the moment of the submission of the manuscripts.

Data on caries will be recorded considering the tooth as the unit of analysis. The progression or change of status can be described with three modalities: 1—as the change from no caries stage to initial stage or to moderate stage or extensive; 2—as the change from initial stage to moderate or extensive stage; and 3—as the change from moderate stage to extensive stage. The net caries increment for initial, moderate and extensive caries levels, using ICDAS (Δ-initial, Δ-moderate and Δ-extensive), will be calculated in primary and permanent molars. Differences between groups in terms of caries increment will be evaluated using the nonparametric Mann–Whitney *U* test. Data analysis will be conducted through Kaplan–Meyer graphs to evaluate caries increment. A comparison of the methods will be carried out with Cox regression with shared frailty. The calculation of sensitivity, specificity and accuracy will consider the results obtained with the indices and the classification of caries lesion presence by the proposed reference standard. The cost-effectiveness ratio will also be verified, considering as effectiveness the prevention of the primary outcome. For all tests, two-tailed analyses will be used, considering a level of significance of 5%. The quality-adjusted life-year index (QALY) will be calculated to assess the value of the preventive intervention [22]. To determine QALYs, the utility value associated with a given state of health by the years lived in that state will be calculated. A year of life lived with no caries increment will be worth 1 QALY (1 year of life × 1 utility value) [22, 23]. Plans to promote participant retention and completion of follow-ups will be performed; a list of any outcome in participants who discontinue the treatment will be compiled and the data will be analysed, taking into account that living conditions in Ladakh do not allow for high mobility of the population. This will make participant retention a minor problem.

The effectiveness of the treatment will be assessed for those who fully followed the protocol (per-protocol subjects). The efficacy and consequences of treatment will be also considered, calculating the event rate (ER) for each group and the number needed to treat (NNT) [24]. An event will be defined as the change of status at tooth level, i.e. the development of a new lesion or the progression of an existing lesion to a more severe level or the change of a tooth previously affected in a filled or a missing tooth during the 2-year follow-up period.

Analyses will be performed using the statistical package Stata 16.0 (Stata Corp, College Station, USA).

### Monitoring

#### Data monitoring

An independent regulation of data collection, management and analysis will be applied independently by the authors.

### Harms

The procedures performed offer minimal risk to patients’ oral health. Side effects of the administration of both chewing gums will be assessed by means of a questionnaire administered to the participants’ parents after 3 months from the beginning of the experimental period and a second time at the end of the administration period.

### Auditing

Data will be stored by one of the authors of the study. Data will be weekly inspected. The inconsistencies will be verified, corrected and registered.

### Ethics and dissemination

#### Research ethics approval, consent and assent

No official ethical committee is present in the area of the survey. The study proposal was already submitted to the authorities of the Zanskar Tibetan Hospital Health Care & Sowa Rigpa Research Institute and its coordinator, the Lama Zopta, for their approval. Approval will also be asked to each school involved in the survey. Due to language difficulties and the population’s low literacy rate, verbal approval will be obtained for children’s participation by their parents or guardians. Plans for communicating important protocol modifications will be prepared and, in case, disseminate them to relevant parties.

#### Confidentiality

Participants will be coded with identification numbers to guarantee confidentiality during data analysis. Participants’ files will be stored in a secure room in the Department of Restorative, Preventive and Pediatric Dentistry, University of Bern.

#### Access to data

Full protocol, clinical trial data and statistical code will be granted full access via public repository after acceptance of the final reports and manuscripts of the trial.

#### Ancillary and post-trial care

Parents/guardians of the children participating in the trial will receive information about the children’s oral status. If caries lesions in dentine will be detected in the children enrolled in the trial, the children will insert in a treatment plan list and they will be contacted for cares by the medical centre in Padum (CHC, community health centre).

#### Dissemination policy

A full report of the findings will be prepared and submitted in full length through national and international journals and newsletters and via website.

## Discussion

Dental caries continues to be one of the most prevalent human diseases worldwide. The actual skewed caries figure suggests the need of implementing effective preventive approaches, especially for high-risk groups such as children living in low socioeconomic contests [25]. The children population living in the remote valley of Zanskar in Ladakh has shown a very high caries prevalence and still no caries preventive programme at the school level has ever been implemented. Due to the lack of dental facilities in the area, a school-based preventive programme seems to be the most effective strategy to reach children. Since caries is a behaviour-related disease, especially in children, different caries-preventive measures have been proposed, such as using sugar substitutes. Sugar-free chewing gums, especially those containing Xylitol, were effectively administered to reduce caries-associated risk factors and caries lesion increment [7–9].

A randomized controlled trial is considered the most powerful study design, in which at least two interventions are contemporaneously administered to two or more groups of subjects forming the arms of the trial. The random enrolment into each arm reduces the potential risk of bias [25]. Randomization is generally performed at an individual level. However, a group-randomized design may be useful in situations where there is a presumed lack of feasibility of carrying out the intervention at the individual level or/and when researchers aim to obtain findings on the intervention effect at the group level, as it happens with the school-based intervention [26]. School-based programmes are effective strategies to improve oral health among schoolchildren, particularly among underprivileged groups of children at high caries risk [25, 27]. Promoting children’s health through schools is strongly recommended by the World Health Organization [28].

Just one recent paper assesses the salivary concentration of xylitol released from two chewing gums containing different amounts of the polyol in a sample of healthy volunteers [29] so the proposed trial will be first clinical trial aiming to evaluate and understand the use of chewing gums with Xylitol as the only sweetener. To the best of the authors’ knowledge, this will be the first clinical trial to assess the effect of a sugar-free Xylitol chewing gum on caries prevention in a population with special living conditions like that of Ladakh. The hypothesis under evaluation is that there will be a statistically significant difference between the interventions regarding caries increment. The findings will contribute to strengthen the evidence for the efficacy of Xylitol use in community-based caries prevention programmes in children.

## Trial status

The trial is not recruiting participants yet. The recruitment will start when the COVID-19 pandemic will allow it.

## Supplementary Information

**Additional file 1.** SPIRIT checklist.

## Data Availability

The dataset will be available in a public repository after the acceptance of the manuscripts.

## Abbreviations

Xyl: Xylitol group
Pol: Polyol group
dmft: Decayed, missed, filled teeth (primary dentition)
DMFT: Decayed, Missed, Filled Teeth (permanent dentition)
ICDAS: International Caries Detection and Assessment System Codes
QALY: Quality-adjusted life-year index
